# A Novel Approach to Pharmacy Practice Law Instruction

**DOI:** 10.3390/pharmacy9020075

**Published:** 2021-04-03

**Authors:** Matthew Deneff, Lisa M. Holle, Jill M. Fitzgerald, Kathryn Wheeler

**Affiliations:** 1 Consultant Pharmacist, Lake Oswego, OR 97035-1575, USA; matthew.deneff@gmail.com; 2 Department of Pharmacy Practice, University of Connecticut School of Pharmacy, 69 North Eagleville Road, Storrs, CT 06269-3092, USA; jill.fitzgerald@uconn.edu (J.M.F.); kathryn.wheeler@uconn.edu (K.W.)

**Keywords:** simulation, pharmacy skills, laws, dispensing, pharmacy education

## Abstract

Pharmacy law instruction is often taught as a didactic course; however practical application of pharmacy law is a main component of pharmacy practice. Technology-based simulations are becoming more frequently used to enhance didactic pharmacy education. The goal of this study was to evaluate the utility of and student perceptions on the usefulness of MyDispense community pharmacy simulation for additional law instruction that if successful might prompt curricular revamping. This Institutional Review Board–approved, two-year, qualitative, prospective, survey study was conducted in a case study class where students completed MyDispense exercises focused on common legal issues that arise in practice, both individually before and within groups during class. Participating students completed a qualitative survey directed at use of MyDispense for pharmacy law review, which included a series of close-ended questions graded on a Likert scale and open-ended questions thematically grouped. Thirty-eight (41%) and twenty-eight (31%) students completed surveys in 2017 and 2018, respectively. The majority of respondents felt exercises improved their understanding of pharmacy laws, focused on challenging areas, and were more interesting than additional lectures. However, certain topics were reported as irrelevant based on practice experiences or not ideal for simulation, and students desired exercises on state laws versus pharmacy policies. Students reported the MyDispense simulation exercises helped them to recall pharmacy laws and focus on topics that were challenging. These study results prompted curricular revamping to incorporate MyDispense throughout the curriculum for practice in recognizing and solving legal scenarios, along with didactic course changes.

## 1. Introduction

The Multistate Pharmacy Jurisprudence Examination (MPJE) is one of two exams to be licensed to practice pharmacy. Pharmacists must pass the MPJE to demonstrate mastery of the legal components of pharmacy practice for licensure in a participating jurisdiction [1]. It is a mixed-format exam with multiple choice, ordered response, and multi-response questions pertaining to a combination of state and federal pharmacy laws. Examination centers on the MPJE Competency Statements, three distinct groupings (“Areas”) of laws impacting practice. Area 1, Pharmacy Practice, encompasses legal aspects of prescription dispensing, counseling, and recordkeeping; personnel roles and responsibilities; and general day-to-day functions of a pharmacist. Licensure, Registration, Certification, and Operational Requirements comprise Area 2, which includes licensing requirements for personnel and pharmacies as well as requirements for operation within a licensed entity. Area 3, General Regulatory Processes, covers application of laws “that regulate or affect manufacture, storage, distribution, and dispensing of pharmaceutical products, preparations, bulk substances/excipients, and devices, including controlled substances.”

Students in the University of Connecticut (UConn) School of Pharmacy previously received formal law education in the spring semester of the second professional (P2) year. However, students reported that they felt unprepared for the MPJE both before and after graduation during routine informal feedback sessions with curricular assessment faculty (personal communication, Kathryn Wheeler). Data from 2009 to 2016 MPJE testing indicated a significant downward trend in scores for Connecticut, relative to the national average [2]. The 2009 average MPJE score for in-state UConn candidates was 94.12, trending down to 76.06 in 2016. By comparison, the national average from 2009 was 93.94, only dropping to 83.77 in 2016. These scores stabilized between 2017–2020 (during the time the study was conducted) for UConn in-state candidates (76.71–77.58), while scores dropped even further for national candidates, 78.39–78.75.

The negative trend in graduate performance on the MPJE for licensure in both Connecticut and other jurisdictions indicates an unmet need for additional or novel methods of teaching pharmacy law. At the time this study was conducted (2017–2020), UConn pharmacy students received instruction on law for one semester in the second professional year, then took the MPJE after completion of the fourth professional year and graduation. No additional formal lecture or follow-up on legal topics were regularly incorporated into the curriculum until students were provided an examination review course at the time of graduation.

Curricular review in this area not only addresses the needs of future graduates, but also can be used to meet pharmacy education Accreditation Council for Pharmacy Education (ACPE) accreditation standards in the United States [3]. In particular, Standard 10.11, curriculum review and quality assurance, specifies that curricular design and delivery are evaluated regularly and revised when appropriate. Additionally, Standard 19.1 for educational effectiveness states, “Faculty members have the capability and demonstrate a continuous commitment to be effective educators and are able to effectively use contemporary education techniques to promote student learning in all offered pathways.” The overall intention of the ACPE standards is to promote continuous growth, improvement, and innovation in pharmacy education. Ideally, graduates are enabled to apply knowledge through critical and innovative thinking, while faculty are expected to adapt new teaching methods to provide the best possible learning opportunities for the required curriculum.

One novel and potentially more effective teaching method is the use of technology-aided simulations. Simulation is utilized in a high percentage of medical and nursing programs and has been increasingly incorporated into pharmacy education [4]. Previous studies have shown “an increase in the areas of student knowledge, self-perceived clinical skills, […] patient safety awareness, and interprofessional teamwork skills” [5]. Vyas and colleagues [5] quantified the extent of use of standardized patients and high-fidelity mannequins in pharmacy curricula in the United States. Their assessment showed that although simulation-based teaching methodologies are in use by many schools of pharmacy, ample room for future growth exists. Since that review publication, several studies have evaluated the use of simulation to enhance learning of a specific topic (eg, nephrology pharmacotherapeutics, advanced cardiac life support) showed improved learning outcomes with simulation [6,7] None, however, had used simulation to enhance learning pharmacy practice law.

Thus, based on the success of simulation in other curricular areas, we decided to incorporate simulation into our curriculum to enhance learning of pharmacy law and evaluate its use. We chose to use MyDispense, a web-based, customizable, community pharmacy simulation program originally developed by faculty at Monash University in Melbourne, Australia, which has further been adapted for use in the United States [8,9]. MyDispense simulates a community pharmacy experience to enable students to complete exercises on a personal computer. The system allows students to practice the skills associated with dispensing medications, including asking questions of patients and providers, interpreting and filling prescriptions, and providing patient counseling. It provides a safe environment for students to practice therapeutic and clinical skills without endangering patients and is accessible on any computer. It has been implemented in several pharmacy education programs globally and demonstrated successful outcomes in various courses including community dispensing skills and therapeutics of cardiovascular diseases [10,11]. Furthermore, the customizable and adaptable nature of MyDispense allows for countless instructional opportunities throughout pharmacy education, such as pharmacy practice law [9].

The purpose of this study was to assess the utility and student perceptions of law-related MyDispense simulation activities to enhance learner understanding of pharmacy practice law. Ultimately, the results of this study along with MPJE scores from these learners were used to improve content delivery through curricular revamping.

## 2. Methods

### 2.1. Setting

This two-year, qualitative, prospective study was approved by the UConn Institutional Review Board and took place as a part of a required course, PHRX 5011: Correlated Pharmacy Problem Solving (CPPS). PHRX 5011 was a third professional year integrative course in which students apply knowledge from other courses throughout pharmacy school to case studies and clinical scenarios. It encouraged student participation and teamwork to formulate solutions that were then discussed as a group. This process facilitated critical thinking using prior knowledge to generate possible solutions and follows up with discussion on the solutions generated in order to correct or explain any unclear points. Students were separated into four sections of approximately 20 to 25 students each and were further separated into groups for collaboration within each section.

All students were required to complete a series of MyDispense exercises both prior to and during their assigned course section. Eight pre-class exercises that required the student to determine if the prescription as written was legal to fill (e.g., including laws for controlled substance dispensing, dosage form substitution, and prescriptive authority) as well as scenarios about laws pertaining to inventory and recordkeeping requirements, were utilized for the 2017 session. Based on feedback from the first group of participants, the 2018 session included the same number of exercises with increased focus on exercises that focused on the legal requirements of a prescription, as well as substitution requirements, fraud, and emergency controlled-substance fills. In class, students completed 5 exercises in small groups, which centered on laws and regulations related to human and animal prescriptions (e.g., label requirements, prescription information requirements), emergency filling of controlled substances, patient counseling requirements, and antiepileptic medication substitutions (i.e., inability to automatically substitute products).

Feedback was provided to students on performance in each exercise. Students received immediate scoring of their performance as correct, partially correct, or incorrect through MyDispense, with pre-filled feedback on the appropriate law and reasoning behind the mark given. At the end of each in-class session, students were provided with additional clarification on particularly challenging exercises and any unclear points raised throughout the class. Following completion of the assigned exercises, students were offered the opportunity to participate in this research study.

### 2.2. Research Instrument

A survey was developed to qualitatively evaluate student perceptions of the use of MyDispense within the required course to supplement their learning of pharmacy practice law learned in a previous Law and Ethics course. The first portion of the survey included a series of close-ended questions graded on four- and five-point Likert scales in 2017 and 2018, respectively. A 5-point scale was chosen in 2018 after a literature review revealed that the 5-point scale would make the results more generalizable. These questions focused on the student’s perception of whether MyDispense exercises helped them understand pharmacy practice law, was enjoyable/interesting, relevant and challenging. Open-ended questions were also included to obtain students’ thoughts on the timing of this practice, the content of the exercises, and alterations that could be made to improve the applicability or comprehensiveness of the simulations.

### 2.3. Data

The electronic survey was administered anonymously through Qualtrics^XM^ (Provo, UT, USA). Data collection took place in 2 cohorts: November 2017 (class graduating in May, 2019) and November 2018 (class graduating in May, 2020).

### 2.4. Statistical Methods

Descriptive statistics were used to report the numerical data. Mean Likert scale scores were calculated for the close-ended survey questions. Open-ended question responses were collated, thematically grouped, and reported for each cohort.

## 3. Results

Thirty-eight students (41%) and twenty-eight students (31%) participated in the 2017 and 2018 surveys, respectively. The overall response from those participating in the survey, as well as students who did not complete the survey but attended the session, was positive. Constructive feedback for future improvements was provided. For the 2017 session, results are shown below in Table 1. All 4-point Likert scale scores averaged above the point of neutrality, 2.5. The two most positive sections focused on the usefulness of MyDispense in practicing pharmacy law and the interesting nature of the practice session.

Results from the 2018 session (see Table 2) show all survey scores averaged over 3, the point of neutrality for the 5-point Likert scale. In the 2018 session the most positive responses again included the usefulness of MyDispense in practicing pharmacy law and the interesting nature of the practice session. Students in this session also responded that the simulations helped them recall pharmacy laws and focused on areas that were challenging, more so than in the previous year.

Open-ended qualitative feedback included recommendations for law concepts/scenarios that should have more focus within the exercises (e.g., differentiation between store policies and state and federal laws and regulations, specifics on controlled substances, emergency supply and substitution of dosage forms and brand/generic, how to handle the requirements for recordkeeping); see Supplementary Table S1. Students recommended to spend less focus on validation and error identification scenarios.

## 4. Discussion

This is the first study evaluating the usefulness and student perception of the virtual community pharmacy program MyDispense for practicing application and recall of pharmacy practice laws after completing a pharmacy law and ethics course. The addition of MyDispense practice served to potentially bridge the long time gap between the course and the MPJE by allowing practice of law concepts throughout the curriculum. The results of our student survey indicated a lack of functional understanding of pharmacy practice laws following formal course instruction and the utility of MyDispense simulations as a solution to this problem. While students found the exercises to be challenging, virtual patient simulation provided an innovative way for students to recall and apply laws vital to everyday pharmacy practice. Constructive feedback elucidated opportunities for future growth and improvement of these exercises. Increased emphasis will be placed on controlled substance dispensing, differentiation of federal and state laws from store policy, and documentation requirements, while prescription verification-type exercises will be stressed less.

Our results are similar to other studies, which also evaluated students’ self-reported engagement and satisfaction with virtual patient simulation activities. In studies specifically evaluating MyDispense implementation to supplement classroom material in a variety of courses (e.g., communication, community pharmacy practice, and law and ethics skills development and self-care), students reported a moderate to high value of MyDispense exercises in assisting them with learning the skills related to pharmacy practice within a community-based setting [9,10,12,13]. Only one study evaluating MyDispense in a cardiology therapeutics course reported a negative student perception [11]. However, Shin and colleagues reported that despite this negative student perception, the completion of the optional MyDispense exercises were positively and significantly correlated with exam scores. Recently, MyDispense evaluated student performance related to validation of controlled substances prescriptions during a pharmacy law course at Wingate University School of Pharmacy [14]. Students completed fourteen exercises simulating verification of controlled substance prescriptions in addition to traditional coursework. Students who completed more than one-half of all available exercises had significantly higher scores on the midterm exam compared with those students who did not complete fewer exercises (*β* = 0.28; *p* = 0.02). While further investigation of MyDispense’s effect on retention of practiced material and the impact of prior community pharmacy experience is required, students responded positively to reinforcement of course content through this modality.

Students also have found value in other virtual simulation programs incorporated into pharmacy education [15,16,17,18]. For example, Bernaitis and colleagues [17] employed a branched-narrative virtual simulation program in oncology therapeutics instruction. Students were free to make real-time clinical judgments and reason through problems without danger to patients; each decision led toward an outcome, right or wrong, of the student’s own making. Students had access to the exercises anywhere and were able to complete them as many times as desired after the initial workshop. Additionally, the program provided feedback throughout the simulated scenario. Upon evaluation at the end of the course, overall scores were markedly improved from the previous year where no virtual patient exercises were incorporated (*p* < 0.05), and feedback indicated a strong positive response from students involved. Similarly, a study published by Barnett and colleagues [18] showed that use of a virtual patient case in an osteoarthritis laboratory compared with a paper-based case significantly improved student self-perceived confidence in managing a patient with osteoarthritis (*p* ≤ 0.02) but also demonstrated that approximately two-thirds to three-fourths of students strongly agreed or agreed that completion of the simulated case resulted in increased interest, enjoyment, relevance, and realism, and assisted them with learning new content and was realistic to clinical practice.

### 4.1. Limitations and Future Studies

Similar to previously published literature on the benefits of simulation-based learning in pharmacy education, our study highlights the value of virtual patient simulations for use in a pharmacy law review following formal instruction and prior to licensing review. MyDispense as a platform for review was well-received by students with room for improvements to exercise content and the clarity of answer explanations. However, our study is not without limitations. The data evaluated were qualitative and used for gauging students’ perceptions. While this subjective feedback is valuable when utilizing novel teaching methods and seeking to implement curricular changes, it is unable to show the objective value provided by these changes, which is better illustrated with data on course examination and MPJE averages. The MPJE in-state UConn graduate pass rate averages for these 2 cohorts of students receiving MyDispense law education in 2017 and 2018 were 77.08 (national, 83.77) in 2019 and 76.71 (national, 78.75) in 2020 [2]. Although we used the MPJE scores as the impetus for this study, the applicability of the MPJE pass rate to reflect the multiple changes we made to the course is limited. For example, the questions asked on each MPJE differ, which may reflect differences in year-to-year pass rates. Additionally, the content taught with these MyDispense exercises and within the course may not reflect the full content of MPJE as exact content is unknown by instructors. Furthermore, the national average scores also remained low; possibly reflecting a difference in the MPJE examination content during these years compared to higher scoring years in the last decade (2009–2016). Additionally, the survey response rate in our study was low. Only thirty-eight and twenty-eight students responded in 2017 and 2018, respectively, out of each class of approximately eighty-five students. Finally, our study did not include a corresponding traditional lecture component or comparator group as many previously published reports have; nor did we assess impact of MyDispense on grades within a law course. Further investigation of MyDispense use in legal instruction should include both of these components to strengthen the evidence for its value.

### 4.2. Managerial and Academic Implications

Based on the positive student perception of MyDispense for practicing pharmacy law within this study, the UConn School of Pharmacy undertook a curricular change to more formally include MyDispense in conjunction with lecture-based law education throughout the curriculum. However, we also recognized the identified gap in law knowledge following formal instruction needed additional curricular change. Thus, the pharmacy law course was moved to the 3rd professional year fall semester and was revamped from the previous 1-credit lecture/exam format type course to be a 3-credit course due to the significance of this subject, the implications it has on licensing of future pharmacists, and the additional learning components added. The new curriculum still includes exams (a midterm and final) but they are now administered with Examsoft software (Dallas, TX, USA) that allows each exam item to be mapped to the blueprint of the MPJE to help the instructor ensure coverage of appropriate material and identify areas where students struggle. Active learning components were also included, such as student presentations/projects, use of in-class clicker response technology, and use of the prescription drug monitoring program to address the current opioid epidemic. MyDispense law activities are now used throughout the curriculum in the Skills-based courses to enhance law understanding. As with any curricular change, it is critical to review and perform quality assurance. Evaluation of these curricular changes is an ongoing process and it is anticipated that continual improvement and innovations will be made.

## 5. Conclusions

Use of MyDispense after formal law education was well-received by students when placed between initial didactic education and licensing examination. Students found the simulation exercises helped them to recall pharmacy laws and focus on topics that were challenging. Qualitative feedback from students identified topics that should have focus in future education (e.g., laws and regulations vs. store policies, controlled substance activities). These results prompted curricular revamping to improve upon the content delivered using MyDispense throughout the curriculum for practice recognizing and solving legal scenarios, in addition to formal review sessions.

## Figures and Tables

**Table 1:** Survey responses from 2017 P3 pharmacy students after participation in MyDispense law activities.

Survey Question	Number of Participants (%)				Mean Likert Response
	Strongly Disagree	Disagree	Agree	Strongly Agree	
These exercises helped me recall pharmacy laws (*N* = 38)	2 (5.2)	3 (7.8)	28 (73.7)	5 (13.2)	2.95
These exercises helped me practice my understanding of pharmacy laws (*N* = 38)	2 (5.2)	1 (2.63)	27 (71.1)	8 (2.1)	3.08
I enjoyed this method of reviewing pharmacy laws (*N* = 38)	3 (7.9)	8 (2.1)	18 (47.4)	9 (23.9)	2.87
This method of reviewing pharmacy laws is more interesting than a lecture (*N* = 38)	1 (2.6)	4 (10.5)	19 (50)	14 (36.8)	3.21
This method of reviewing pharmacy laws is more interesting than group discussion of a case (*N* = 38)	3 (7.9)	13 (34.2)	19 (50)	3 (7.9)	2.58
The use of MyDispense is relevant to pharmacy practice laws (*N* = 38)	2 (5.2)	6 (15.8)	25 (65.8)	5 (13.2)	2.87
These exercises focused on areas that were challenging for me (*N* = 38)	1 (2.6)	5 (13.2)	16 (42.1)	16 (42.1)	2.95

**Table 2:** Survey responses from 2018 P3 pharmacy students after participation in MyDispense law activities.

Survey Question	Number of Participants (%)					Mean Likert Response
	Strongly Disagree	Disagree	Neither Agree or Disagree	Agree	Strongly Agree	
These exercises helped me recall pharmacy laws (*N* = 28)	1 (3.57)	2 (7.14)	3 (10.71)	16 (57.14)	6 (21.43)	3.86
These exercises helped me practice my understanding of pharmacy laws (*N* = 27)	0 (0)	4 (14.81)	1 (3.7)	15 (55.56)	7 (25.93)	3.93
I enjoyed this method of reviewing pharmacy laws (*N* = 27)	2 (7.41)	5 (18.52)	6 (22.22)	9 (33.33)	5 (18.52)	3.37
This method of reviewing pharmacy laws is more interesting than a lecture (*N* = 27)	0 (0)	1 (3.7)	4 (14.81)	12 (44.44)	10 (37.04)	4.15
This method of reviewing pharmacy laws is more interesting than group discussion of a case (*N* = 27)	1 (3.7)	7 (25.93)	8 (29.63)	8 (29.63)	3 (11.11)	3.19
The use of MyDispense is relevant to pharmacy practice laws (*N* = 27)	2 (7.41)	2 (7.14)	3 (11.11)	14 (51.85)	6 (22.22)	3.74
These exercises focused on areas that were challenging for me (*N* = 26)	0 (0)	3 (11.54)	4 (15.38)	14 (53.85)	5 (19.23)	3.81

## Data Availability

The data presented in this study may be available on request from the corresponding author. The data are not publicly available due to ethical and privacy concerns.

## Supplementary Materials

The following are available online at https://www.mdpi.com/article/10.3390/pharmacy9020075/s1, Table S1: Thematically selected qualitative feedback from students after participation in MyDispense law activities.
