# Supplementary material for: A Novel Approach to Pharmacy Practice Law Instruction
Source: Pharmacy (Basel). 2021 Apr 3;9(2):75. doi: 10.3390/pharmacy9020075 (PMC8167573; doi:10.3390/pharmacy9020075)
Supplement: Supplementary file 1 [file pharmacy-09-00075-s001.pdf]

Supplemental Table

Table S1. Thematically selected qualitative feedback from students after participation in MyDispense law activities.

What changes do you recommend to the content of these exercises?

- I liked the practice on law out of regular lecture. This makes it easier for us to apply to practice and remember. (2017)
- The MyDispense exercises are a good way to learn pharmacy law to some extent, but the type of exercises that we do could be greatly improved. (2017)
- They may be useful/applicable following the law course post lecture to integrate it into our learning. (2017)
- Differentiate between CT law and National law because a lot of us are not practicing in CT. (2018)
- Add more complex problems for closer to law exam. (2018)
- A tutorial version of these cases where you learn as you go instead of after you finish the entire case may be helpful. This could be followed by cases that we have to do without help. (2018)

List concepts/laws/scenarios that you feel more exercises should be focused on.

- I think the 7-day narcotic supply exercise was a great one because that's a newer law in CT and is something I probably wouldn't have thought to look for. (2017)
- Paperwork laws and federal laws vs state laws; store policy vs laws. (2017-18)
- Emergency supplies for medications. (2017-18)
- Controlled substance dispensing. (2017-18)
- Changing medication routes (oral to solution), substitutions (brand/generic/formulation). (2017-18)

List concepts/laws/scenarios that you feel fewer exercises should be focused on.

- Errors on prescriptions. (2017)
- Checking prescriptions, verification. (2017-18)

Which exercises, if any, were most applicable to issues you have seen in practice, either working or on community rotation?

- Having a drug control person come in and what to have in order for them. (2017)
- The man from the DEA that came and asked about records, honestly. We can find fraudulent scripts easily, but when an official comes in, we are kind of at a loss for what to do. (2017)
- I felt that the best exercises were those that reached into these gray areas that you don't see in practice often but as a pharmacist you certainly need to know. (2017)
- The 7-day restriction on narcotics is realistic. (2017-18)
- Dispensing controlled substances. (2018)
- Emergency supplies with and without controls and tweaking prescriptions to change the route. (2018)
